# Incidence and management of the left ventricular outflow obstruction in patients with atrioventricular septal defects

**DOI:** 10.1093/icvts/ivab303

**Published:** 2021-11-09

**Authors:** Yaroslav Ivanov, Edward Buratto, Phillip Naimo, Adrienne Lui, Thomas Hu, Yves d’Udekem, Christian P Brizard, Igor E Konstantinov

**Affiliations:** 1 Cardiac Surgery Unit, The Royal Children’s Hospital, Parkville, VIC, Australia; 2 Department of Paediatrics, The University of Melbourne, Melbourne, VIC, Australia; 3 Murdoch Children’s Research Institute, Melbourne, VIC, Australia; 4 Melbourne Centre for Cardiovascular Genomics and Regenerative Medicine, Melbourne, VIC, Australia

**Keywords:** Atrioventricular septal defect, Left ventricular outflow tract obstruction

## Abstract

**OBJECTIVES:**

Left ventricular outflow tract obstruction (LVOTO) is a recognized complication after complete repair of atrioventricular septal defect (AVSD). This study reviewed the incidence and management of LVOTO following AVSD repair at a single institution.

**METHODS:**

From 1975 to 2019, 24 patients (3.3%, 24/730) underwent reoperation due to LVOTO following partial AVSD (pAVSD) and complete AVSD (cAVSD) repair. The data were retrospectively reviewed.

**RESULTS:**

The incidence of LVOTO following pAVSD and cAVSD repair was 4.4% (12/275) and 2.6% (12/455). Freedom from LVOTO reoperation following pAVSD and cAVSD repair at 25 years was 94.3% [95% confidence interval (CI); 89.7–96.7] and 95% (95% CI; 91.1–97.3). The median time from complete repair of pAVSD and cAVSD to LVOTO reoperation was 4.4 years [interquartile range (IQR): 3.4–6.7] and 2.6 years (IQR: 2.2–4.7). Freedom from second LVOTO reoperation at 5, 10 and 15 years was 83.7% (95% CI; 57.2–98.2), 59.2% (95% CI; 28.7, 80.3) and 39.5% (95% CI; 13.2–65.3). The median time between the first and the second LVOTO reoperation in the groups of pAVSD and cAVSD was 6.1 years (IQR: 3.4–8.9) and 8.6 years (IQR: 5.7–9.8). There was no significant difference regarding the first (*P* = 0.7406) and subsequent LVOTO (*P* = 0.7153) following complete repair of pAVSD and cAVSD. Combined access to the left ventricular outflow tract was not protective regarding LVOTO reoccurrence. Survival for both groups after LVOTO reoperation at 15 years was 95.6% (95% CI 99.4–72.9).

**CONCLUSIONS:**

Incidence of LVOTO after AVSD repair is low but the reoccurrence rate is high. Standard subaortic resection does not always provide definitive LVOTO relief. The survival after LVOTO reoperation is excellent.

**Figure:**
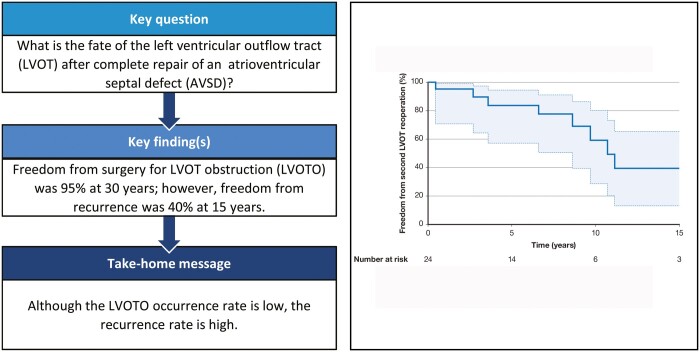

## INTRODUCTION

In the current era, complete atrioventricular septal defect (cAVSD) and partial atrioventricular septal defect (pAVSD) can be repaired with excellent results, however, the burden of reintervention remains significant [1, 2]. Left ventricular outflow tract (LVOT) obstruction is the second most common indication for reoperation, after left atrioventricular valve (LAVV) regurgitation [3, 4]. In the setting of repaired pAVSD and cAVSD, the anatomic nature of the LVOT is complex, it is difficult to predict which patient will develop left ventricular outflow tract obstruction (LVOTO) and, furthermore, it is challenging to address surgically. In this study, we reviewed our experience with the surgical management of LVOTO in the setting of cAVSD and pAVSD. The objective of the study was to describe the incidence of the LVOTO, techniques used for repair and freedom from further reoperations.

## MATERIALS AND METHODS

### Patients

Our institutional database of atrioventricular septal defect (AVSD) was reviewed for all reoperations on the LVOTO.

Data were obtained retrospectively from the hospital patients records and follow-up was conducted via correspondence with the patients’ cardiologists and general practitioners. Follow-up was 100% complete.

### Ethical statement

The study was approved by the Royal Children’s Hospital (RCH) Human Research Ethics Committee (HREC32047E). The requirement for individual patient consent was waived due to the retrospective nature of the study.

### Operative technique

Our surgical approach to complete repair of pAVSD and cAVSD has previously described in detail [1, 2]. Briefly, complete repair is performed via a median sternotomy on full cardiopulmonary bypass at moderate hypothermia. Repair is performed through a right atriotomy, and a 2-patch approach is routinely used, with either expanded polytetrafluoroethylene patch (W.L. Gore and Associates Inc, Newark, DE, USA) or glutaraldehyde treated autologous pericardium used for the ventricular septal defect component. Fresh autologous pericardium was used for the ostium primum. The decision to close the cleft or not was based on echocardiographic and intraoperative assessment of the valve.

**Figure 1:**
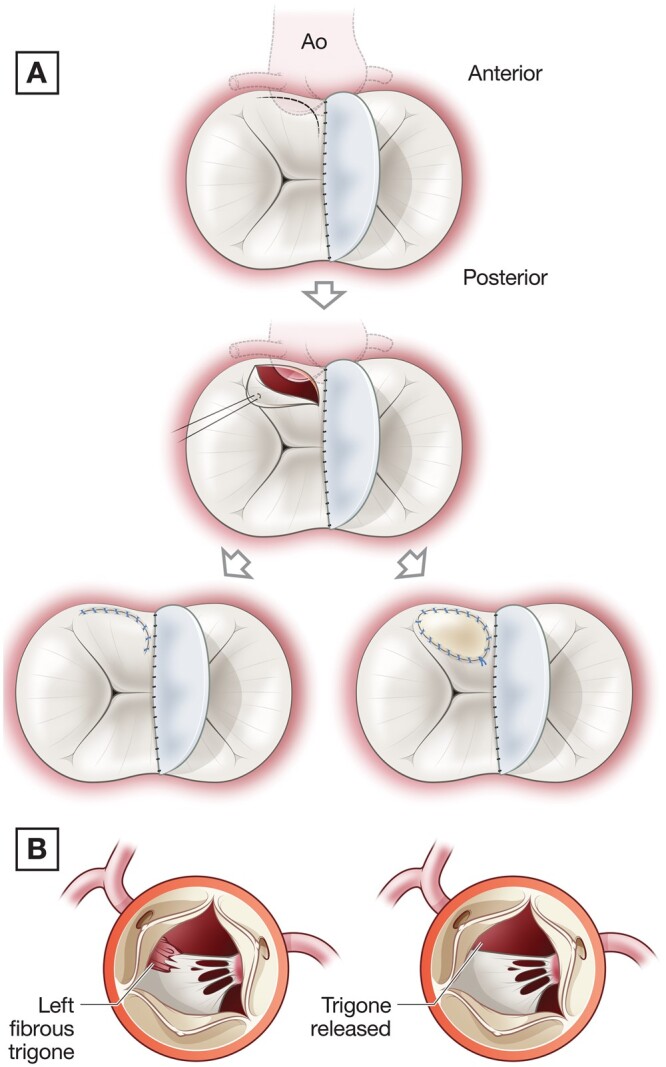
(**A**) Left atrium approach to the left ventricular outflow tract obstruction with superior bridging leaflet augmentation. (**B**) Left fibrous trigone release.

The indication for LVOTO reoperation was peak gradient across the LVOT of 30 mmHg or more. We rely on the combination of transthoracic and transoesophageal echocardiography for planning and immediate postoperative assessment in all cases. All reoperations for LVOTO were conducted via median sternotomy on cardiopulmonary bypass with mild hypothermia. Depending on the anatomical substrate of the LVOTO, the decision was made intraoperatively how it should be approached. The surgical approaches were as follows: transaortic, left atrial or combined approach. A fibromuscular membrane was present in the vast majority of cases and consequently, it was approached via the aorta. The technique of fibromuscular resection has been previously described [5]. The left atriotomy approach was required for complex cases of LVOTO when the anatomical substrate was accessory cords, valvular tags or diffusely narrowed LVOT. By retracting the left aspect of the superior bridging leaflet upwards, the LVOT was inspected and, if needed, the accessory cords and tags were resected. In instances, where the LVOT was problematic to visualize from the left atrium, the anteroseptal aspect of the superior bridging leaflet was detached from the annulus which provided unrestricted access to the LVOT. In cases with diffusely narrowed LVOT when the cause of the LVOTO was small anteroposterior dimensions of the LVOT, the left aspect of the anterior bridging leaflet required augmentation with the glutaraldehyde treated pericardial patch or CardioCel patch (Admedus Regen Pty Ltd, Perth, WA, Australia) (Fig. 1A). In addition, to normalize the hinge mechanism function between the interventricular septum and the left aspect of the superior bridging leaflet, the left fibrous trigone was released. By releasing of the fibrous trigons, we imply sharp resection of the fibrous nodule in the area of the left fibrous trigone. In addition, we resect sharply all basal cords or additional small papillary muscles in this area (Fig. 1B).

### Statistics methods

All data were analysed using STATA version 13 (Stata Corp, College Station, TX, USA). Continuous data are expressed as median and interquartile range (IQR). Fischer exact test was used comparing discrete variables when group size was <5. Time-dependent endpoints, specifically survival and freedom from LVOTO operation, were analysed using the Kaplan–Meier method, with time commencing at the time of initial AVSD repair. Differences between groups were compared using the log-rank test. The threshold for statistical significance was taken as *P *<* *0.05.

## RESULTS

### Incidence

From 1975 to 2019, a total of 275 patients with pAVSD underwent complete repair at the RCH, Melbourne, Australia. From 1990 to 2019, a total of 455 patients with cAVSD underwent complete repair in the RCH. One patient with concomitant double outlet right ventricle and pulmonary atresia was also included to the study group. A total of 24 patients (3.3%, 24/730) underwent reoperation for LVOTO in the study period. Of the 730 patients who underwent repair for cAVSD and pAVSD, 3.2% (24/730) required reoperation on the LVOT during the study period.

**Figure 2:**
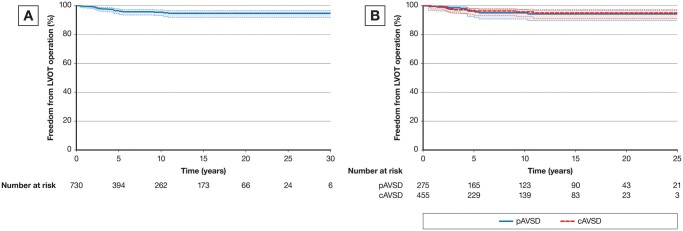
(**A**) Freedom from the left ventricular outflow tract obstruction reoperation for the total group of 730 patients. (**B**) Freedom from left ventricular outflow tract obstruction reoperation in groups of cAVSD and pAVSD. cAVSD: complete atrioventricular septal defect; pAVSD: partial atrioventricular septal defect.

The freedom from LVOTO operation for the total group of 730 patients after cAVSD and pAVSD repair at 5, 15 and 30 years was 96.7% (95% CI; 94.6, 97.9), 94.8% (95% CI; 92.1, 96.7) and 94.8% (95% CI; 92.1, 96.7), respectively (Fig. 2A).

The incidence of LVOTO in the group of patients after cAVSD repair was 2.6% (12/455). All 12 patients with cAVSD had 2-patch technique. The median time from the complete repair to the first LVOTO reoperation was 2.6 years (IQR: 2.2–4.7). A total of 12 patients required 16 reoperations due to LVOTO. Out of them, 41.7% had initially Rastelli type A of cAVSD (5 out of 12); 25% (3 out of 12) had Rastelli type C of cAVSD; 8.3% had Rastelli type B of cAVSD and in the rest 3 (25%) patients the type of cAVSD was not recorded at the time of the complete repair. Overall, the freedom from LVOTO reoperation in the group of cAVSD repair at 5, 15 and 25 years was 96.7% (95% CI; 94, 98.4), 95% (95% CI; 91.1, 97.2) and 95 (95% CI; 91.1, 97.3), respectively (Fig. 2B).

The incidence of LVOTO in the group of patients after pAVSD repair was 4.4% (12/275). The median time from pAVSD repair to the LVOTO reoperation was 4.4 years (IQR: 3.4–6.7). A total of 12 patients underwent 16 reoperations due to LVOTO; 4 patients (33.3%) out of 12 required second LVOTO resection due to LVOTO reoccurrence. At the time of the first LVOTO operation, 4 patients (33.3%) underwent concomitant LAVV repair. Freedom from LVOTO operation for partial AVSD at 5, 15 and 25 years was 96.4% (95% CI; 94.2, 98.2), 94.3% (95% CI; 89.7, 96.9) and 94.3% (95% CI; 89.7, 96.9), respectively (Fig. 2B).

### Surgical management

The median of mean and peak LVOT gradient at the first LVOTO operation was 40 mmHg (IQR: 34–44) and 65 mmHg (IQR : 43–78) and for the second LVOTO reoperation, they were 60 mmHg (IQR: 52–62) and 92 mmHg (IQR: 78–100), respectively.

A total of 32 LVOTO reoperations were performed on 24 patients with complete and partial AVSD. The median cardiopulmonary bypass time was 73 min (IQR: 53–129) while the mean cross-clamp time was 37 min (IQR: 29–69), respectively. For patients undergoing a second LVOTO operation, the median bypass time was 120 min (IQR: 73–157) and the median cross-clamp time was 85 min (IQR: 54–112).

The anatomical substrate for the LVOT obstruction is represented in Table 1.

**Table 1:** The anatomical substrate of the LVOT obstruction

Anatomical substrate	The first LVOT operation, (*n* = 24, %)	The second LVOT operation, (*n* = 8, %)
Fibromuscular membrane	23 (96%)	8 (100%)
Additional cords	9 (35%)	0
Valvular tags	3 (12.5%)	0
Diffuse and complex narrowing of the LVOT	2 (8.3 5%)	0

LVOT: left ventricular outflow tract.

**Figure 3:**
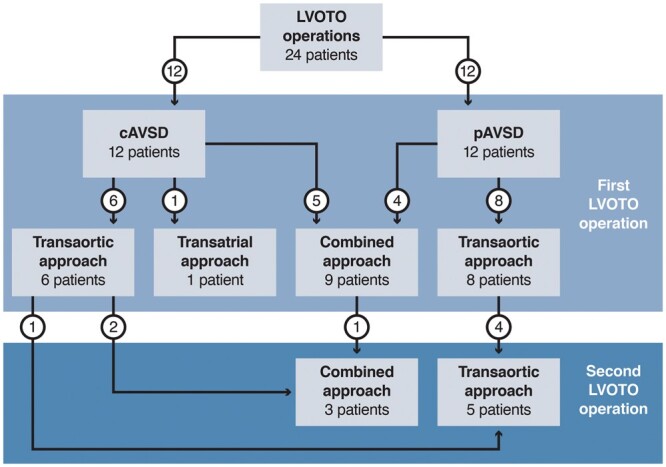
Surgical approaches to the left ventricular outflow tract obstruction. cAVSD: complete atrioventricular septal defect; LVOTO: left ventricular outflow tract obstruction; pAVSD: partial atrioventricular septal defect.

The surgical approaches to LVOTO are presented in Fig. 3.

The summary of the surgical details is represented in Table 2.

**Table 2:** The surgical details regarding LVOTO operations

Surgical details	The first LVOTO operation (*n* = 24, %)	The second LVOTO operation (*n* = 8, %)
Fibromuscular membrane resection with myotomy	18 (75%)	6 (75%)
Fibromuscular membrane resection without myotomy	5 (20%)	2 (25%)
Additional cords resection	9 (37.5%)	
Superior bridging leaflet augmentation	4 (16.7%)	2 (25%)
Left fibrous trigone release		3 (37.5%)
Valvular tags resection	3 (12.5%)	0
Ventriculoplasty	1 (4.2%)	

LVOTO: left ventricular outflow tract obstruction; VSD: ventricular septal defect.

Surgical details of 2 patients with complex and diffuse narrowing of the LVOT are presented below. One patient with cAVSD and associated double outlet right ventricle with pulmonary atresia required left ventricular rerouting (tunnelling) using a segment of the 12-mm Gore-Tex tube. Later, he developed LVOTO due to the outgrowth of the ventricular septal defect patch, so it was replaced with bigger piece of the Dacron conduit 10.7 years after complete repair. Another patient after partial cAVSD repair underwent LVOTO operation 16 months later which included augmentation of LVOT by means of superior bridging leaflet augmentation with autologous pericardium and ventriculoplasty in addition to conventional subaortic membrane resection and myotomy.

### Follow-up

The median follow-up time in the group of partial AVSD repair was 14.8 (IQR: 3.8–22.2) years. One patient died in the group of pAVSD due to bowel cancer at age of 18 years; 90.7 months after the LVOT reoperation. In the group of patients with complete AVSD, the median follow-up was 12.9 years (IQR: 4.3–4.7). There was no late death in this group.

**Figure 4:**
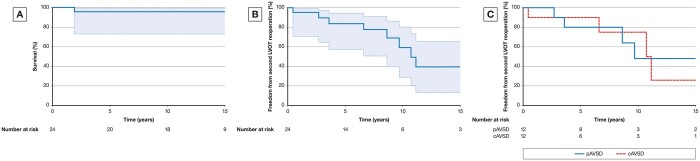
(**A**) Survival. (**B**) Freedom from the second left ventricular outflow tract obstruction reoperation in the combined group of patients. (**C**) Freedom from the second left ventricular outflow tract obstruction operation. cAVSD: complete atrioventricular septal defect; pAVSD: partial atrioventricular septal defect.

Overall, the survival for both groups after LVOTO reoperation at 5, 10 and 15 years was 95.6% (95% CI 99.4; 72.9%) (Fig. 4A).

The freedom from the second LVOTO reoperation for the total group of 24 patients at 5, 10 and 15 years was 83.7% (95% CI; 57.2, 98.2), 59.2% (95% CI; 28.7, 80.3) and 39.5% (95% CI; 13.2, 65.3), respectively (Fig. 4B). Between groups with complete repair of cAVSD and pAVSD, there was no significant difference with regards to first (*P* = 0.7406) and subsequent LVOT (*P* = 0.7153) reoperations.

Of 12 patients with cAVSD who required LVOTO reoperation, 4 (33.3%) required the second LVOTO reoperation due to its reoccurrence. The median time from the first LVOTO reoperation to the second LVOTO reoperation was 8.6 years (IQR 5.7–10.8). At the time of the first LVOTO reoperation, 16.7% of patients (2 out of 12) required concomitant LAVV repair. None of the patients needed LAVV repair at the time of the second LVOTO reoperation. The freedom from the second LVOTO operation after the first LVOTO reoperation at 5, 10 and 15 years was 90% (95% CI; 47.3, 98.5), 75% (95% CI; 29.8, 93.4) and 25% (95% CI; 10.4, 65.7), respectively (Fig. 4C).

Of the 12 patients with the first LVOTO repair after complete pAVSD repair, 4 required second LVOTO repair with the median time in between the first and the second LVOTO operation of 6.1 years (IQR: 3.4–8.9). The freedom from the second LVOTO operation after the first LVOTO reoperation at 5, 10 and 20 years was 80% (95% CI; 40.9, 94.6), 48% (95% CI; 11.2, 77.6) and 48% (95% CI; 11.2, 77.6), respectively (Fig. 4C).

### Left atrioventricular valve function after superior bridging leaflet intervention

In total, there were 6 patients who underwent superior bridging leaflet detachment with subsequent patch augmentation of the resulting opening. There were 4 and 2 patients on whom this was performed as a first and second LVOTO procedure, respectively. The patch material we used for the closure of the opening was as follows: untreated autologous pericardium patch (*n* = 2), glutaraldehyde treated autologous pericardium (*n* = 2) and Cardiocell patch (*n* = 2). All 6 patients have mild LAVV regurgitation at the time of presentation with LVOTO. After LVOTO operation in 2 of them, the amount of regurgitation remained unchanged and in another 4 it decreased to trivial in 3 and to no regurgitation in 1. However, after 1 year, 1 patient was reoperated for severe LAVV insufficiency due to tearing suture line on the superior bridging leaflet patch. After reoperation, the amount of the LAVV insufficiency became trivial as it was immediately after LVOTO repair.

## DISCUSSION

Although it is well documented that complete repair of cAVSD and pAVSD is associated with low early mortality between 1.4% and 2.9% [2, 6, 7] for cAVSD and 0.8–4.5% [1, 8, 9] for pAVSD, there remains a relatively high burden of reoperations in groups. It is also well established that the most common reoperations in both groups are due to residual LAVV incompetence and LVOTO [7, 10]. Our group has previously identified rate and modality of presentation of reoperations in groups of complete repair of pAVSD [1] and cAVSD [2], however, more detailed study regarding LVOTO after complete repair of AVSD have not been performed. Thus, the incidence and surgical management of LVOTO in patients who underwent complete repair of cAVSD and pAVSD with focus on incidence, reoccurrence and surgical management were reported.

Anatomical factors which provide the substrate for the development of LVOTO include reduced inlet and increased outlet portion of the left ventricle leading to an elongated and narrowed LVOT, abnormal anchoring of superior bridging leaflet contributing to the tightness of the subaortic area, and deficiency of inlet portion of the interventricular septumare present in up to 70% patients after complete AVSD repair [11–14]. Interestingly, those patients with repaired pAVSD could be up to 3 times more prone to the development of LVOTO [14]. Although the highest reported incidence of LVOTO in cases of pAVSD was 22.5% [15], in the vast majority of reports the incidence was up to 5% [8, 16, 17] in both groups of pAVSD and cAVSD. In our series, the incidence of LVOTO in groups of pAVSD and cAVSD was 4.4% and 2.6% which is comparable with reports from other institutions when incidence of LVOTO was reported between 1.2% and 5.3% [6, 8, 16, 17]. Comparing previous reports from our institution the incidence of LVOTO remains largely unchanged for both groups of patients [2, 10]. Median time from complete repair in groups of pAVSD and cAVSD to LVOTO reoperation was 4.4 years and 2.6 years, which is generally consistent with reports of other groups [8, 17]. Interestingly, that Ginde *et al.* reported median time to LVOTO reoperation of 6.2 years in the group of cAVSD repair. Despite the fact that the incidence of LVOTO presentation was higher in the pAVSD group compared with the cAVSD group, the freedom from LVOTO reoperation at 25 years was almost identical at around 95%. This finding probably suggests that irrespective of the underlying anatomy (cAVSD versus pAVSD) and surgical approach (double patch technique for cAVSD versus single patch technique for pAVSD), the ‘anatomical’ substrate is similar after complete repair with identical time-related risks for developing LVOTO. This is further supported by the recent findings by Fong *et al.* [7] comparing the outcomes of modified single patch repair versus double patch repair for cAVSD, given that the modified single patch technique alters the anatomy of cAVSD to a state which is analogous to repaired pAVSD [18]. By doing propensity score matching between 2 surgical strategies, the freedom from LVOTO reoperation was not significantly different (*P* = 0.64).

**Figure 5:**
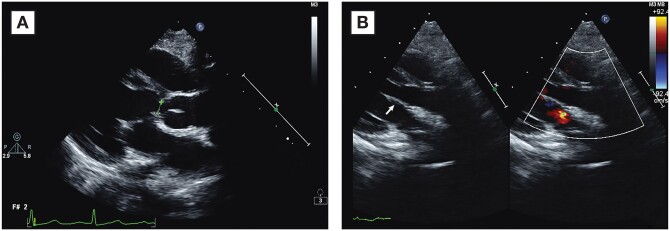
(**A**) Left ventricular outflow tract before superior bridging leaflet augmentation. (**B**) Left ventricular outflow tract after superior bridging augmentation. The arrow indicates the patch on the superior bridging leaflet.

We observe unexpectedly low freedom from recurrent LVOTO reoperations at 25% at 15 years in the group of cAVSD and 48% at 20 years in the group of pAVSD giving an overall 39.5% freedom from recurrent LVOTO reoperation at 15 years. A similar trend was observed by Stulak *et al.* [18] who reported freedom further reoperations on the LVOT in the group of pAVSD at 96% at 5 years and 70% at 10 years. Another report by Van Arsdell *et al.* [19] observes 6-year freedom from recurrent LVOTO at 66% ± 15% in a mixed group of AVSD. These findings reflect complex nature of LVOTO as an evolving disease and prompted utilization more than conventional surgical strategies to deal with it. The predominant anatomical source of LVOTO in vast majority patients (96%; 23/24) at the time of first LVOTO reoperation and in all cases of recurrent LVOTO reoperation was fibromuscular membrane. In all but one case, it was approached through the aorta. Additional cords and valvular tags usually do not generate significant gradient in the LVOT but considering that LVOT is longer than in normal heart they could cause turbulence contributing to LVOTO development thus should be resected when present [11]. In our series, they were present in around one-quarter of patients with LVOTO occurrence with no patients having them at the time when LVOTO reoccurred. We tend to resect them via an LA approach utilizing superior bridging leaflet detachment as a ‘window’” to the proximal LVOT (Fig. 1A). Intuitively, we can expect that utilizing the combined access at the time of the first LVOTO repair might reduce the risks of LVOTO reoccurrence, but we did not observe that (*P* = 0.4). Malattachment of the superior bridging leaflet to the ‘scooped-out’ crest of the ventricular septum that leads to narrowing of proximal LVOT is another target for LVOTO relief [19–21]. Anatomically, this abnormal attachment predisposes to the displacement of the superior bridging leaflet towards LVOT producing obstruction (Fig. 5A). By means of augmenting superior bridging leaflet, the LVOT can be made shorter and wider (Fig. 5B). In our series, 25% (2/8) of patients with recurrent LVOTO required superior bridging leaflet augmentation reflecting the fact that fibromuscular resection is not always sufficient to relieve LVOTO. This is in line with finding Stulak *et al.* [4] who also found that ∼25% of patients who underwent fibrous resection or septal myectomy at first reoperation required more complex surgery which in their series was Konno operation. The resection of the fibrous trigones is relatively new technique for the LVOTO operation that was described for the non-AVSD patients [22, 23]. The application of this technique was limited to resection of the left fibrous trigone leaving the right trigone unattended. We used this technique in the instances of the recurrent LVOT obstruction.

### Limitations

This study is limited by the retrospective nature and small number of patients. Due to the long study period, some echocardiographic data were not routinely reported.

## CONCLUSION

Incidence of LVOTO after AVSD repair is low but reoccurrence rate is high. Standard subaortic resection does not always provide definitive LVOTO relief. Thus, other anatomical targets of LVOTO should be addressed. Superior bridging leaflet augmentation should be considered in addition to conventional subaortic resection.

## Funding 

P.N. is supported by a National Health and Medical Research Council Medical Research Postgraduate Scholarship (1150242). Y.d’U. is a Practitioner Fellow of the National Health and Medical Research Council of Australia (1082186).

**Conflict of interest:** Prof Yves d’Udekem is a consultant for Actelion and MSD. A/Prof Christian Brizard serves on the advisory board of Admedus.

### Data Availability Statement

All relevant data are within the manuscript and its supporting information files.

### Author contributions

**Yaroslav Ivanov:** Conceptualization; Data curation; Project administration; Writing—original draft; Writing—review & editing. **Edward Buratto:** Data curation; Formal analysis; Methodology; Writing—review & editing. **Phillip Naimo:** Data curation; Writing—review & editing. **Adrienne Lui:** Data curation. **Thomas Hu:** Data curation. **Yves d’Udekem:** Writing—review & editing. **Christian P. Brizard:** Project administration; Supervision; Visualization; Writing—review & editing. **Igor E. Konstantinov:** Conceptualization; Project administration; Supervision.

### Reviewer information

Interactive CardioVascular and Thoracic Surgery thanks Mark H.D. Danton, Carl Lewis Backer and the other anonymous reviewers for their contribution to the peer review process of this article.

## ABBREVIATIONS

AVSD: Atrioventricular septal defect
cAVSD: Complete atrioventricular septal defect
CI: Confidence interval
IQR: Interquartile range
LAVV: Left atrioventricular valve
LVOT: Left ventricular outflow tract
LVOTO: Left ventricular outflow tract obstruction
pAVSD: Partial atrioventricular septal defect
